# Ornithine Transcarbamylase Deficiency Presenting as Acute Encephalopathy After Strabismus Surgery

**DOI:** 10.7759/cureus.26667

**Published:** 2022-07-08

**Authors:** John Lung, Sunil Sathappan, Isra Sabir, Richard Maier

**Affiliations:** 1 Internal Medicine, University of Nevada Reno School of Medicine, Reno, USA; 2 Critical Care Medicine, Renown Health, Reno, USA

**Keywords:** critical care and internal medicine education, ornithine, urea cycle disorder, altered mental state, acquired ornithine transcarbamylase deficiency

## Abstract

Acute encephalopathy with an unclear etiology is a common presentation to the hospital. We describe the case of a 50-year-old male who presented with a one-day history of slurred speech, nausea, insomnia, and altered mental status. His surgical history was notable for a strabismus surgery two days ago. He presented with elevated ammonia levels that continued to increase. Metabolic studies were suggestive of hyperammonemia secondary to ornithine transcarbamylase (OTC) deficiency triggered due to fasting prior to the strabismus surgery. OTC gene sequencing confirmed the diagnosis of OTC deficiency. We summarize the current case reports in the literature and review the treatment options for OTC deficiency. Our case occurred after a low-risk outpatient strabismus surgery and is a good example of maintaining a broad differential and revising the suspected diagnosis constantly.

## Introduction

Acute encephalopathy with an unclear cause is a common presentation to an emergency department. Imaging, basic labs, and a good history usually lead the treatment team to a preliminary diagnosis during the first few days of admission. Occasionally, patients can quickly decline to a comatose state that requires intubation. In cases where the patient has an elevated ammonia level, the differential can be either related to overproduction of ammonia or reduced elimination of ammonia. More common causes include drug-induced hyperammonemia, liver failure, starvation, urease-producing bacteria, and gastrointestinal hemorrhage. Less common causes include metabolic errors that include urea cycle disorders [1]. Though less common, untreated hyperammonemia caused by urea cycle disorders leads to escalating complications, including cerebral edema, structural damage of the brain, and death [2]. The most common subtype of urea cycle disorders is ornithine transcarbamylase (OTC) deficiency. Overall estimates of urea cycle disorders are approximately one in 35,000, with the most common subtype of OTC deficiency at approximately one in 63,000 [3]. We present the case of a patient who presented with acute metabolic encephalopathy with headaches, required intubation within 24 hours of admission, and was later diagnosed with OTC deficiency with his hyperammonemia exacerbated by multiple factors.

## Case presentation

A 50-year-old male presented to the emergency department with a one-day history of slurred speech, nausea, insomnia, and altered mental status. He reported no fevers, cough, or headaches. He denied any alcohol, drug, or tobacco use. His surgical history was notable for a strabismus surgery two days ago to correct diplopia. His medical history was remarkable for Reye’s syndrome at age seven. Initial laboratory workup was notable for sodium of 145 mmol/L, chloride of 112 mmol/L, CO_2_ of 21 mmol/L, and anion gap of 12 mmol/L. Initial imaging studies including computed tomography (CT) of the head without contrast, CT angiogram of the head with and without contrast, and magnetic resonance imaging (MRI) of the brain without contrast were unremarkable. The patient was admitted and started on intravenous (IV) antibiotics and antiviral medication, including ceftriaxone, acyclovir, and ampicillin. Due to continued agitation, an ammonia level was ordered that was elevated at 260 µg/dL. His chemistry panel now had a sodium of 150 mmol/L, chloride of 116 mmol/L, CO_2_ of 17 mmol/L, and anion gap of 17 mmol/L. His hepatitis serologies were negative, and an abdominal ultrasound was unremarkable. Due to worsening mentation and respiratory alkalosis on arterial blood gas (pH: 7.60, pCO_2_: 20 mmHg, pO_2_: 85 mmHg, HCO_3_: 19 mmol/L), the patient was intubated for airway protection. He was started on lactulose and rifaximin. Subsequently, he was transferred to our facility for a higher level of care.

**Figure 1:**
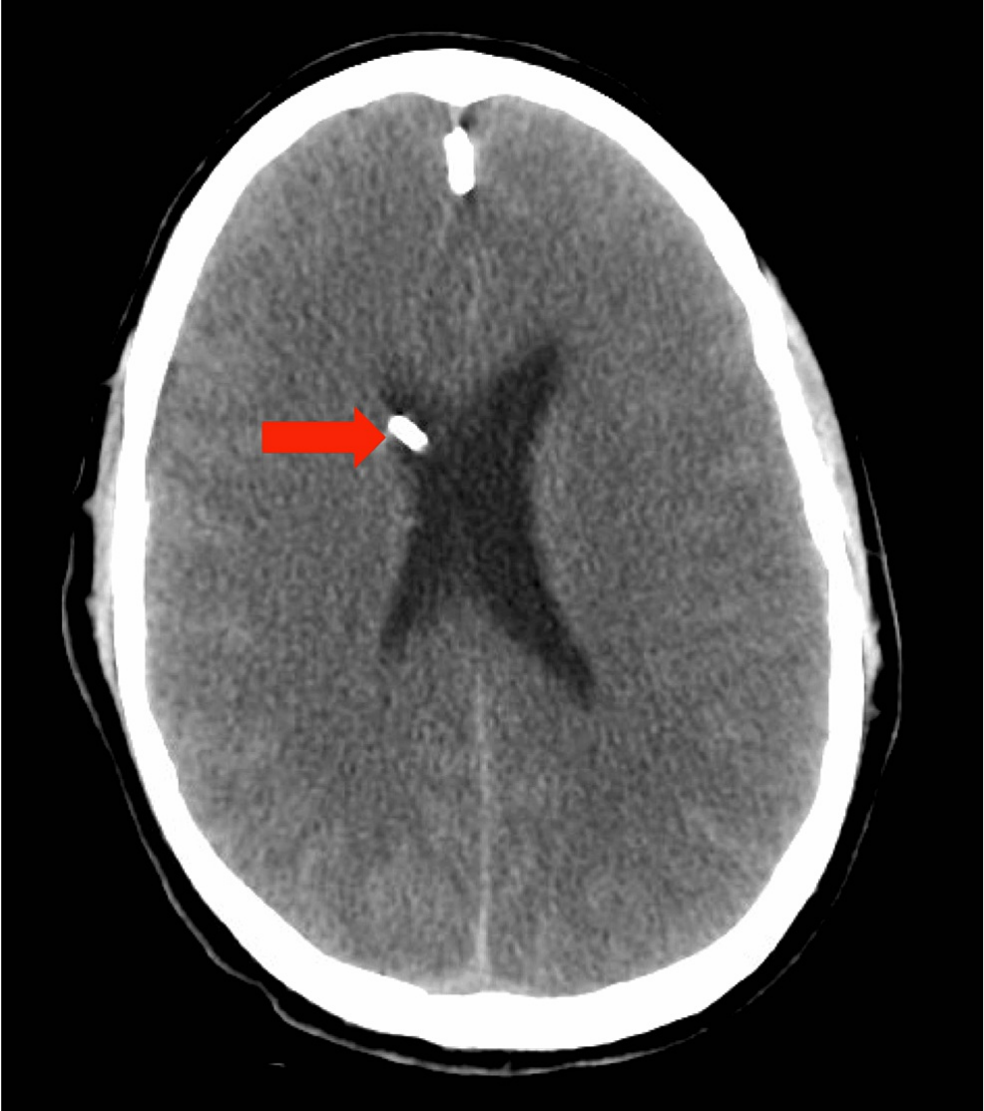
Computed tomography of the head without contrast showing right frontal external vascular drain placement (red arrow). Also evident is diffuse loss of gray/white differentiation.

Once transferred, nephrology was consulted for emergent dialysis. The patient’s ammonia level after arrival was 608 µg/dL. During placement of the dialysis catheter, the patient had status epilepticus that was treated with lorazepam and propofol. Serial head CT scans were suggestive of possible development of cerebral edema. Nephrology performed emergent hemodialysis and recommended IV furosemide. Due to concern of a urea cycle disorder, the patient was administered ornithine, citrulline, and sodium benzoate. These interventions brought the patient’s ammonia down to 46 µg/dL. Our facility did not have the standard treatment for urea cycle disorders of phenylacetate and sodium benzoate available. The patient was transferred to another facility the next day because it was unclear if the hyperammonemia had fully resolved or if ammonia would continue to accumulate. Before transfer, central venous access was obtained and antibiotics covering urease-producing bacteria were given, including ceftriaxone, azithromycin, and clindamycin. A right frontal external vascular drain with an opening pressure of 30 cmH_2_O was placed by neurosurgery for worsening cerebral edema (Figure 1). The opening pressure improved to 4 cmH_2_O after external vascular drain placement.

At the receiving quaternary care center, genetics and hepatology were consulted for workup of rare metabolic disorders. Hepatology ordered an ultrasound of the abdomen, reviewed the previous workup, and agreed that the profound hyperammonemia was more consistent with an inborn error of metabolism versus acute or chronic liver disease. Genetics was consulted which ordered a workup of plasma amino acids, urine organic acids, plasma acylcarnitine, alpha-ketobutryate, cerebrospinal fluid (CSF) amino acid study, OTC deficiency gene sequencing, and deletion/duplication genetic study. Genetics also started arginine, citrulline, levocarnitine, enteral feeds without protein, IV sodium phenylacetate, and sodium benzoate. Further history obtained by the genetics team revealed Reye’s syndrome at age eight with encephalopathy after a viral illness and acute encephalopathy at age 32 with a similar presentation to this admission that was diagnosed as transient global aphasia. Due to the patient’s worsening cerebral edema, hypertonic therapy with mannitol and hypertonic saline was started. The patient also developed a left lower extremity deep vein thrombosis (DVT) on hospital day six and was anticoagulated with heparin drip then switched to apixaban. On hospital day nine, the patient was extubated. On hospital day 10, the right frontal external vascular drain was removed. Later in the hospitalization on day 25, the patient developed a worsening DVT in the left gastrocnemius and soleal veins. He was switched to enoxaparin. The metabolic studies found elevated glutamine in plasma and CSF, low citrulline in CSF, and high orotic acid in urine. Genetics concluded that the patient had hypomorphic OTC deficiency that was triggered by a catabolic state due to fasting prior to the strabismus surgery, followed by eating a high protein diet with tofu and fish the day before and after the surgery. The fasting and high protein diet caused hyperammonemia and triggered his altered mental status and coma. He was advised to limit protein intake, avoid steroids, and avoid prolonged fasting. Genetics recommended that he continue citrulline, levocarnitine, and sodium phenylbutyrate. He was discharged to an inpatient rehabilitation facility on hospital day 28. After discharge, the OTC sequencing and deletion/duplication sequencing was hemizygous for the OTC c.903A>T;p.Leu301Phe variant, consistent with OTC deficiency.

## Discussion

The diagnosis of OTC deficiency is supported by suggestive clinical findings, including encephalopathic episodes, recent stress, Reye-like syndrome, unexplained cerebral palsy, and a history of recurrent vomiting. The diagnosis is established by a hemizygous pathogenic variant of OTC by molecular genetic testing, abnormal increase in orotic acid excretion after an allopurinol challenge test, or decreased OTC enzyme activity in the liver [4]. OTC deficiency typically occurs in males as it is X-linked. Our patient had a Reye’s episode as a child, an unexplained encephalopathy episode in adulthood, and a recent stress event of fasting before his strabismus surgery. He was also hemizygous for a pathogenic variant of OTC deficiency (p.Leu301Phe), a single-point mutation found in previous cases [5].

**Figure 2:**
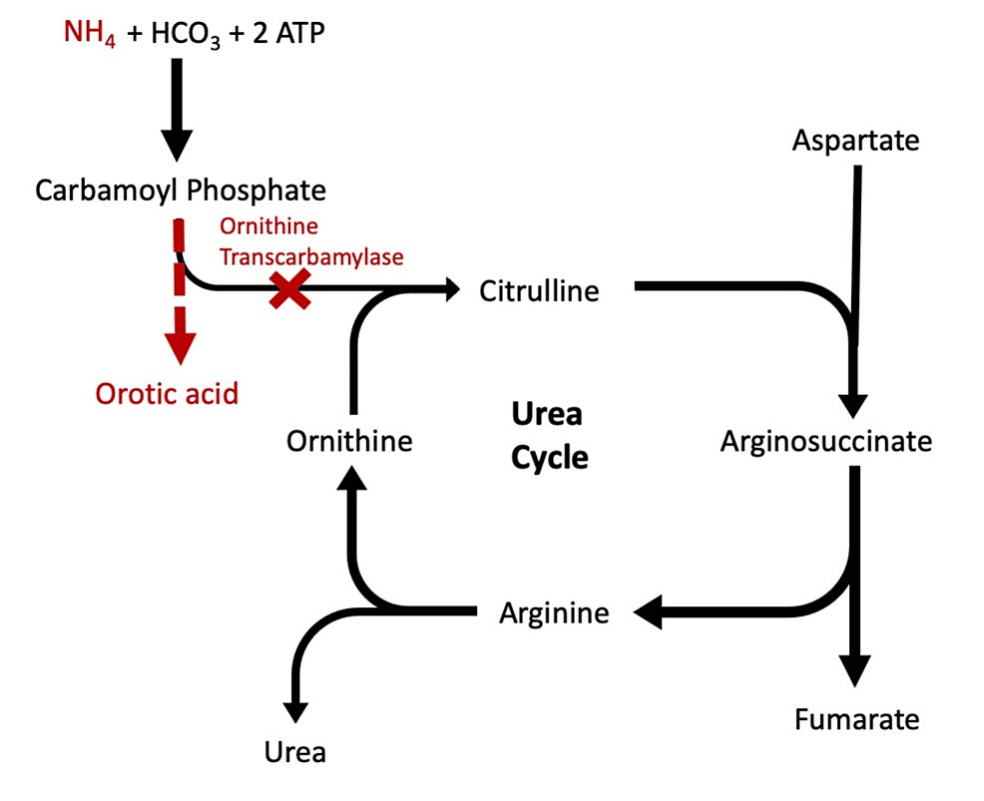
OTC deficiency disrupts the excretion of nitrogen through the urea cycle leading to the accumulation of ammonia and orotic acid (in red). OTC: ornithine transcarbamylase

OTC deficiency leads to increased levels of circulating ammonia in the body as all organs generate ammonia during metabolism. The deficiency of OTC prevents ammonia clearance through the urea cycle (Figure 2). Ammonia can easily cross the blood-brain barrier, and high levels of ammonia are toxic to brain tissue [6].

The major goal of the treatment of hyperammonemia secondary to OTC deficiency is to lower ammonia levels. Emergent hemodialysis can be performed if ammonia levels are higher than 200 µg/dL. Sodium benzoate and sodium phenylacetate should be administered to eliminate ammonia alternative pathways that bypass the urea cycle [7]. Any protein in the diet should be stopped for at least 48 hours, and then restarted to avoid further protein catabolism. Administration of arginine reduces urea levels, and administration of carnitine provides cerebral protection [8]. After acute hospitalization, patients should follow up with a metabolic specialist who recommends dietary changes, such as protein restriction, and nitrogen scavenger therapy, such as phenylbutyrate and citrulline. Liver transplantation is a curative therapy but is only considered in patients with recurrent hyperammonemia that fails medical management [4].

A review of the literature found 23 cases of adult-onset OTC deficiency [8-13]. Of these cases, only two occurred postoperatively after a renal transplant or liver transplant [14,15]. Our case occurred in a low-risk outpatient strabismus surgery, which has not been reported in the literature. The stress of fasting for the surgery combined with increased protein intake before and after the surgery revealed his previously unknown OTC deficiency. These factors lead to a catabolic state causing his encephalopathy and comatose state.

## Conclusions

Our case illustrates the rare occurrence of OTC deficiency after a low-risk surgical procedure not reported previously in the literature. Hyperammonemia has a very broad differential with more frequent causes other than OTC deficiency. However, if there is no clear etiology, clinicians should consider alternative rare diagnoses such as a urea cycle disorder. Clinicians can then obtain further history from the patient or family members such as a history of encephalopathy increasing the suspicion of a urea cycle disorder, including OTC deficiency. Unexplained hyperammonemia should be treated quickly with emergent hemodialysis and transfer to a facility capable of management of inborn metabolic disorders. Further workup for OTC deficiency can be completed when the patient is stable. Our case is a good example of maintaining a broad differential and revising the suspected diagnosis, especially if the patient continues to decline with current therapy modalities.
